# Longitudinal Phospho-tau217 Predicts Amyloid Positron Emission Tomography in Asymptomatic Alzheimer’s Disease

**DOI:** 10.14283/jpad.2024.134

**Published:** 2024-07-24

**Authors:** Robert A. Rissman, M. C. Donohue, O. Langford, R. Raman, S. Abdel-Latif, R. Yaari, K. C. Holdridge, J. R. Sims, D. Molina-Henry, G. Jimenez-Maggiora, K. A. Johnson, P. S. Aisen, R. A. Sperling, A4 and LEARN Study teams

**Affiliations:** 1 Alzheimer’s Therapeutic Research Institute, Keck School of Medicine of the University of Southern California, San Diego, CA USA; 2 Eli Lilly and Company, Indianapolis, IN USA; 3 Brigham and Women’s Hospital, Massachusetts General Hospital, Harvard Medical School, Boston, MA USA; 4 Department of Physiology and Neuroscience, USC Alzheimer’s Therapeutic Research Institute, 9880 Mesa Rim Road, San Diego, CA USA

**Keywords:** PET, p-tau, immunoassay, longitudinal amyloid, Alzheimer’s disease

## Abstract

**Background:**

Blood-based AD biomarkers such as plasma P-tau217 are increasingly used in clinical trials as a screening tool.

**Objectives:**

To assess the utility of an electrochemiluminescence (ECL) immunoassay in predicting brain amyloid PET status in cognitively unimpaired individuals.

**Setting:**

Plasma samples collected at baseline, week 12, and week 240 or endpoint originated from the Anti-Amyloid Treatment in Asymptomatic Alzheimer’s Disease (A4) trial and the companion Longitudinal Evaluation of Amyloid Risk and Neurodegeneration (LEARN) study.

**Participants:**

Both A4 and LEARN enrolled eligible cognitively unimpaired persons 65 to 85 years. Individuals with elevated brain amyloid PET levels were eligible for the A4 Study, while those without elevated brain amyloid PET levels were eligible for the LEARN Study.

**Intervention:**

Participants in the A4 Study received intravenous solanezumab (up to 1600 mg) or placebo every 4 weeks. The LEARN Study is an observational study without intervention.

**Measurements:**

Plasma P-tau217 concentration levels from A4 Study participants were measured using an ECL immunoassay. Receiver Operating Characteristic (ROC) curve analysis was performed for each biomarker against amyloid positivity, defined by ≥22 CL and ≥ 33 CL.

**Results:**

Receiver operating characteristic curve (ROC) analysis indicates high diagnostic value of P-tau217 in individuals with amyloid PET ≥ 20 (Area under the ROC (AUROC): 0.87) and ≥ 33 CL (AUROC: 0.89). Repeated testing with the placebo group taken 12 weeks apart (range: 68 to 143 days) and the LEARN participants taken between 1.4 and 1.75 years resulted in a strong positive correlation (Corr. 0.91 (0.90 to 0.92)).

**Conclusion:**

An ECL immunoassay testing plasma P-tau217 accurately predicts amyloid PET positivity in cognitively unimpaired individuals. Our future analyses aim to determine if use of this assay may reduce the screening burden of preclinical individuals into anti-amyloid clinical trials.

**Electronic Supplementary Material:**

Supplementary material is available in the online version of this article at 10.14283/jpad.2024.134.

## Introduction

The neuropathological changes observed in Alzheimer’s disease (AD), an age-related neurodegenerative disease, begins at least two decades prior to overt cognitive symptoms. In the US, approximately 6.5 million individuals are living with symptomatic AD, and this is projected to increase to 7.2 million by 2025 (1). AD is characterized by increased cerebral amyloid-beta (Aβ) accumulation that precedes cognitive impairment and phosphorylated tau (P-tau) that increases in direct correlation with cognitive change (2). Cognitively normal individuals with high levels of brain Aβ have more rapid rates of cognitive decline and have higher risk of progressing to overt dementia compared with individuals without elevated brain Aβ (3)

Anti-amyloid therapeutics directed at reducing cerebral Aβ accumulation have demonstrated efficacy in early symptomatic AD populations and are currently being studied in cognitively unimpaired individuals with evidence of AD pathology. If a treatment is approved for preclinical AD, there will be a significant demand for a cost-effective and scalable diagnostic for early detection of elevated brain Aβ (4). Readily available, high throughput tests that can detect the extent of cerebral Aβ plaques and tau pathology using positron emission tomography (PET) and/or cerebrospinal fluid (CSF) have been demonstrated to be effective tools in the clinical trial setting for identification of individuals with AD pathology. It is well established that elevated brain Aβ PET and lower CSF Aβ42/Aβ40 levels in cognitively unimpaired individuals are consistent with AD and identify populations who are at risk of cognitive decline (5–7). CSF studies demonstrate that hyperphosphorylation on specific residues of tau can inform on AD pathology and predict disease progression (8, 9). While these biomarker tools have been invaluable, the invasive nature of the testing in addition to high cost and participant burden has dramatically limited broad use (10–12). Blood biomarkers have been investigated as a screening tool to identify preclinical AD study participants to reduce reliance on CSF and/or PET testing. Because blood sample testing is minimally burdensome and less costly than CSF and PET, use of blood biomarkers may be well suited to identify people with preclinical AD (13, 14).

Measurement of Aβ and tau in blood products, serum and plasma, has dramatically improved in recent years with the implementation of mass spectrometry (MS). Data from the AHEAD study team and others have demonstrated that plasma Aβ42/Aβ40 ratio as well as tau phosphorylated at residue 217 (P-tau217) as measured by MS predicts amyloid PET status with a high degree of sensitivity and specificity (15). The predictive capability of plasma P-tau217 and Aβ42/Aβ40 has been replicated using separate cognitively unimpaired cohorts and all consistently demonstrate that P-tau217 was highly predictive of which participants would develop MCI (16). Recent work using immunoassays supports the use of P-tau217 as an accurate marker for Aβ PET positivity and AD progression (17, 18). Plasma P-tau217 is currently being used as an inclusion criterion in a preclinical AD clinical trial (19).

In this study we used biobanked plasma samples from the active and placebo arms of the completed negative A4 clinical trial, which tested solanezumab. In addition, we included participant samples from the Longitudinal Evaluation of Amyloid Risk and Neurodegeneration Study (LEARN) cohort, a group of individuals determined to be amyloid PET negative, but would have otherwise met A4 inclusion criteria, who were followed in parallel with A4. The A4 trial used amyloid PET to identify eligible participants using the centiloid (CL) metric, which allows for standardization of amyloid PET (20, 21). Although there is no consensus in the field regarding absolute threshold, previous work suggest that CL ≥ 33 reliably identifies individuals with substantial presence of amyloid plaque in brain and enriches for participants who will continue to have accumulation (22–25). As mentioned above, our previous work using MS assays of P-tau217 as a biomarker for amyloid PET positivity were highly successful, in this study we tested whether an electrochemiluminescence (ECL) immunoassay developed by Eli Lilly and Company could yield comparable results and have utility and reliability to predict brain amyloid PET status in cognitively unimpaired individuals.

## Methods

The A4 Study methods have been described previously (26). LEARN was a companion observational study of individuals without elevated brain amyloid but would have otherwise met A4 inclusion criteria. Quantification of P-tau217 was assayed on an analytically validated ECL immunoassay using an MesoScale (MSD) Sector S Imager 600 MM at the CAP-accredited, CLIA-certified Lilly Clinical Diagnostics Laboratory on plasma samples from baseline, week 12, and week 240 or endpoint from A4, and from LEARN. Solanezumab, placebo, and LEARN groups are summarized with means and standard deviations for continuous measures; and counts and percentages for binary or categorical variables (Table 1). Associations among biomarkers at baseline are visualized with scatter plots, locally estimated scatterplot smoothing curves, and Spearman’s rank correlations with 95% confidence intervals. The utility of plasma P-tau217 for predicting amyloid PET level ≥ 20 or 33 CLs is summarized with receiver operating characteristic (ROC) curves and the area under the ROC curve (AUROC) with 95% confidence intervals. The repeatability of P-tau217 is summarized with Spearman’s rank correlation between the first two visits in A4 placebo group and LEARN participants. Observations from A4 placebo group participants which occurred more than 143 days apart were excluded from the repeatability analysis, as were observations from LEARN participants which occurred more than 1.75 years apart.

**Table 1:** Demographics of A4 and LEARN cohort with baseline P-tau217

	**Placebo (N=562)**	**Solanezumab (N=540)**	**LEARN (N=524)**	**Total (N=1626)**
Age (y)	71.9 (5.0)	72.0 (4.6)	70.5 (4.3)	71.5 (4.7)
Female sex	343 (61.0%)	313 (58.0%)	322 (61.5%)	978 (60.1%)
Education (y)	16.5 (2.9)	16.6 (2.7)	16.7 (2.6)	16.6 (2.7)
Racial categories				
White	529 (94.1%)	511 (94.6%)	490 (93.5%)	1530 (94.1%)
Black or African American	14 (2.5%)	10 (1.9%)	12 (2.3%)	36 (2.2%)
Asian	13 (2.3%)	9 (1.7%)	11 (2.1%)	33 (2.0%)
American Indian or Alaskan Native	0 (0.0%)	1 (0.2%)	5 (1.0%)	6 (0.4%)
More than one race	3 (0.5%)	5 (0.9%)	5 (1.0%)	13 (0.8%)
Unknown or Not Reported	3 (0.5%)	4 (0.7%)	1 (0.2%)	8 (0.5%)
Ethnicity				
Not Hispanic or Latino	539 (95.9%)	518 (95.9%)	503 (96.0%)	1560 (95.9%)
Hispanic or Latino	18 (3.2%)	16 (3.0%)	17 (3.2%)	51 (3.1%)
Unknown or Not reported	5 (0.9%)	6 (1.1%)	4 (0.8%)	15 (0.9%)
Family history of dementia (parent or sibling)	430 (76.5%)	395 (73.1%)	348 (66.4%)	1173 (72.1%)
APOE Genotype				
Missing	0	0	2	2
E2/E2	0 (0.0%)	1 (0.2%)	4 (0.8%)	5 (0.3%)
E2/E3	32 (5.7%)	27 (5.0%)	65 (12.5%)	124 (7.6%)
E2/E4	20 (3.6%)	13 (2.4%)	10 (1.9%)	43 (2.6%)
E3/E3	201 (35.8%)	191 (35.4%)	337 (64.6%)	729 (44.9%)
E3/E4	264 (47.0%)	264 (48.9%)	104 (19.9%)	632 (38.9%)
E4/E4	45 (8.0%)	44 (8.1%)	2 (0.4%)	91 (5.6%)
FBP SUVr	1.3 (0.2)	1.3 (0.2)	1.0 (0.1)	1.2 (0.2)
FBP Centiloid	65.6 (32.1)	66.7 (33.5)	4.3 (12.5)	46.2 (40.2)
P-tau217 (U/ml)	0.27 (0.15)	0.29 (0.17)	0.15 (0.05)	0.23 (0.15)
Total A-Beta1-42 (pg/ml)				
Missing	419	420	524	1363
Mean (SD)	815.3 (334.0)	836.0 (319.7)	-	824.8 (327.1)
Total A-Beta1-40 (pg/ml)				
Missing	418	420	524	1362
Mean (SD)	11803.9 (3667.4)	12358.1 (3839.0)	-	12055.8 (3749.4)
Total CSF A-Beta42/40				
Missing	419	420	524	1363
Mean (SD)	0.071 (0.027)	0.071 (0.025)	-	0.071 (0.026)
PACC	−0.0 (2.6)	0.0 (2.8)	0.8 (2.3)	0.3 (2.6)
LM Delayed Recall				
Missing	1	0	0	1
Mean (SD)	12.7 (3.5)	12.6 (3.9)	13.5 (3.4)	12.9 (3.6)
MMSE	28.8 (1.3)	28.8 (1.3)	29.0 (1.2)	28.9 (1.2)

The treatment effect of solanezumab on plasma P-tau217 and amyloid PET CL in A4 is estimated by a constrained longitudinal data analysis model with fixed effects for time, time-by-treatment, age, and APOEe4 status (27). Time is modeled using natural cubic splines with two degrees of freedom (28). Residuals are assumed to be correlated with a heterogeneous Toeplitz variance-covariance structure. The modeled difference between groups is summarized with the nominal (i.e. without multiplicity adjustment) 95% confidence interval and p-value.

Associations among annual change in biomarkers are visualized with scatter plots, linear regression trend lines by treatment group, and Spearman’s rank correlations with 95% confidence intervals. Estimates of change per year were derived from linear models with fixed effects for time, treatment, time-by-treatment, age, and APOEe4 status; and participant-specific random intercepts and slopes. The fixed effects for time were again modeled with natural cubic splines with two degrees of freedom. All analyses were conducted using R version 4.3.2 (29), and the nlme and ggplot2 packages (29). Results will be presented using modelled marginal means and 95% confidence intervals instead of the raw values to reflect the multivariable model utilized used to describe the trend over time. This approach does interpolate the estimates where data are sparse or do not exist. See supplementary Figure 1 for raw scores for P-tau and PET over time.

## Results

Table 1 summarizes the characteristics of the randomized A4 treatment groups (placebo and solanezumab) and LEARN. The overall age of the study population was 71.5 years, 60.1% female and predominantly white (94.1%). On average, compared to LEARN, A4 participants had greater amyloid PET burden (FBP Centiloid: 65.6 (placebo) and 66.7 (solanezumab) vs 4.3 (LEARN)), greater plasma P-tau217 (P-tau217 × 1000: 266.6 (placebo) and 286.9 (solanezumab) vs 147.3 (LEARN)), and higher rate of APOEε4 carriage (Percent E3/E4: 47.0% (placebo) and 48.9% (solanezumab) vs 19.9% (LEARN)). At the time of the writing of this manuscript, CSF Aβ has not yet been assayed in the LEARN cohort.

**Figure 1:**
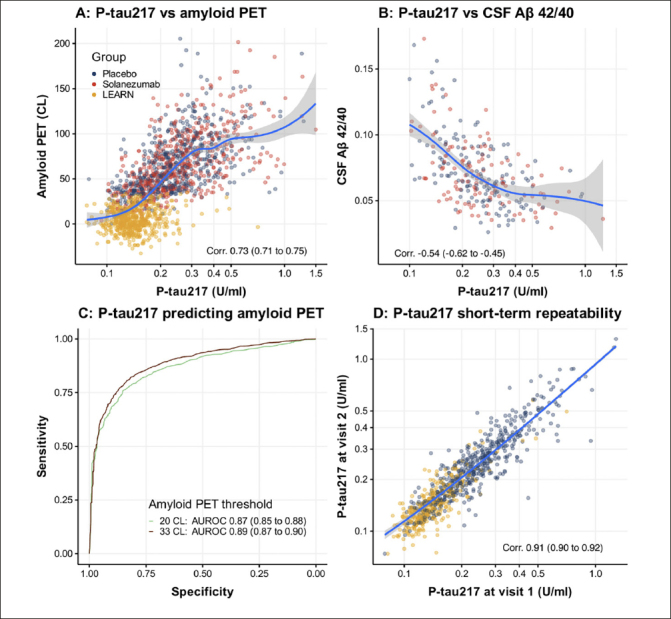
Baseline P-tau217 associations and short-term repeatability Plasma P-tau217 is correlated with amyloid PET centiloids (CL) Panel A) and CSF Aβ 42/40 ratios (Panel B). Receiver operating characteristic (ROC) curves and the area under the ROC curve (AUROC) demonstrate the value of P-tau217 for predicting individuals with amyloid PET greater than 20 or 33 CL (Panel C). Panel D shows short-term repeatability among Placebo group individuals taken 68 to 143 days apart and among LEARN individuals taken between 1.4 and 1.75 years apart. Plasma P-tau217 is plotted with the axis log transformed. Correlations are Spearman’s rank correlations. Values in parentheses are 95% confidence intervals.

Figure 1 demonstrates that plasma P-tau217 is correlated with amyloid PET CL (Corr. 0.73 (0.71 to 0.75) (95% confidence interval); Panel A) and CSF A3B2 42/40 ratios (Corr. -0.54 (-0.62 to -0.45); Panel B). ROC and AUROC demonstrate the value of P-tau217 for predicting individuals with amyloid PET ≥ 20 (AUROC 0.87 (0.85 to 0.88)) or 33 CL (AUROC 0.89 (0.87 to 0.90)) (Panel C). The repeated testing based on observations among placebo group individuals taken 68 to 143 days apart, and among LEARN participants taken between 1.4 and 1.75 years demonstrated good correlation (Corr. 0.91 (0.90 to 0.92)).

**Figure 2:**
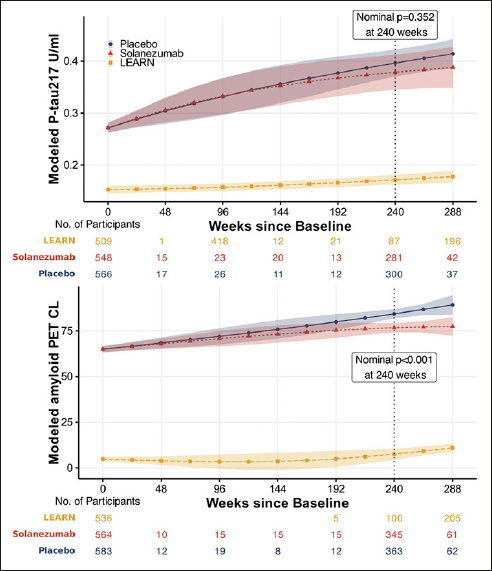
Modeled mean P-tau217 and amyloid PET Natural cubic spline modeling was used to estimate means, 95% confidence intervals and p-values of P-Tau217 (top) and amyloid PET (bottom). Models assume a natural cubic spline for time with two degrees of freedom per group and control for age and APOEε4 carriage; and assume heterogeneous unstructured variance-covariance. Shaded regions are 95% confidence intervals. The p-value is associated with the treatment group difference at 240 weeks, which is indicated by the vertical dotted line. Please note the sparse data in A4 (and no data in LEARN) between baseline (week 0) and week 240, with interpolated estimates in modelled curves.

Figure 2 shows no significant difference between the solanezumab and placebo arms with numerically less accumulation of plasma P-tau217 in the solanezumab treated arm whereas amyloid PET CL showed a similar difference but was statistically significant. The difference between groups at week 240 in P-tau217 was -0.018 U/ml (−0.055 to 0.019; nominal p=0.352). The difference between groups at week 240 in amyloid PET was −7.6 CL (−10.3 to −4.94; nominal p<0.001).

**Figure 3:**
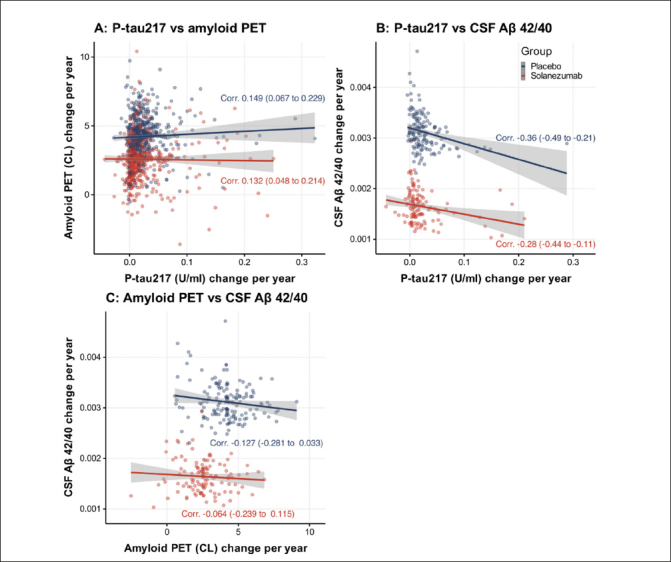
Change in plasma P-tau217 per year P-tau217 is weakly correlated with amyloid PET centiloid (CL) change per year (Panel A), and moderately correlated with and CSF Aβ 42/40 change (Panel B). Correlation between amyloid PET and CSF Aβ 42/40 change is also minimally correlated (Panel C). Estimates of change per year are derived from linear models with fixed effects for time (spline with two degrees of freedom), treatment, time-by-treatment, age, and APOEe4 status; and participant-specific random intercepts and slopes. Correlations are Spearman’s rank correlations of the participant-specific estimates of annualized change at 240 weeks. Values in parentheses 95% confidence intervals. Trend lines are from ordinary least square regression and shaded regions are 95% confidence intervals.

Figure 3 shows change in plasma P-tau217 per year is weakly correlated (about 0.15) with amyloid PET CL change per year (Panel A), and moderately negatively correlated (about −0.3) with and CSF Aβ 42/40 change (Panel B). Correlation between amyloid PET and CSF Aβ 42/40 change is minimal (about −0.1 or weaker, Panel C).

## Discussion

In this study we used longitudinally collected plasma samples from the A4 Study in preclinical AD to assess the utility of an ECL immunoassay for plasma P-tau217. We included a cohort of participants from the A4 Study, which included asymptomatic, high-risk individuals with amyloid plaque levels ≥33 CL. Our findings demonstrate excellent performance for identifying amyloid PET positive, asymptomatic participants through measurement of plasma P-tau217. In addition, we found excellent pharmacodynamic biomarker tracking to amyloid PET change and short-term test repeatability when participant samples from different time-points were compared.

Our data support previous studies using bioassays that target P-tau species to identify staging of AD. Several of these studies demonstrate that elevated plasma P-tau181 correlates with amyloid PET status in MCI and AD cases (9, 31). Other studies report that plasma P-tau217 performs better than P-tau181 for predicting AD pathology (32–34). Furthermore, longitudinal studies using P-tau217 demonstrate increased levels with disease progression (17). Using the P-tau217 ECL immunoassay developed by Eli Lilly and Company, we demonstrated that P-tau217 can be used to predict presence of amyloid neuritic plaque as defined by amyloid PET CL ≥ 20 and 33 which is supported by other recent studies using biosamples from participants with a high degree of family history of AD and APOEε4 allele representation (32). In our analysis, 59% of A4 participants, and 22% of LEARN participants, had at least one APOEε4 allele (Table 1), and 608/766 = 79.4% of APOEε4 carriers were characterized as amyloid PET positive (≥33 CL). MS-based P-tau217 studies have found similar AUC values to what we report here but require somewhat complicated algorithms that include additional variables such as non-phosphorylated tau217, age, BMI and APOEε4 (35). Current biomarkers used for AD screening include the extensively studied P-tau181 in the CSF, which has resulted in reducing screening failures by 50% (36). Recent studies have shown P-tau217 has higher sensitivity over P-tau181 for detecting AD in CSF (37–39). Additionally, plasma P-tau217 performs equally as CSF P-tau217 in detecting AD status, further supporting the use of plasma P-tau217 as a biomarker for AD (40).

Limitations of our study include the sample participants’ racial composition; a high proportion of A4 participants reported being non-Hispanic White (93.8%, Table 1). As we recently published, A4 screen failed Hispanic and non-White participants at a higher frequency during screening, despite a higher prevalence of AD in racial groups such as African Americans and Hispanics/Latino(s) compared to non-Hispanic Whites (41). Although the influence of covariates such as demographics, lifestyle and cognition (5, 42, 43) can be factored into biomarker statistical analyses, the impact of race and/or ethnicity continues to remain elusive. Expansion of cohorts to include racial and ethnic representation among clinical trial participants would provide crucial information (44). With expanded efforts to increase representation of racial and ethnic groups in current trials (e.g. AHEAD, TB3) through use of remote blood screening events and other expanded recruitment efforts, the ability to evaluate potential racial differences in biomarker performance may be possible. Repeat testing across multiple time points may also introduce unaccounted biological variables such as fasting state and time elapsed before freezing. While our model accounts for variables such as time and age, it is possible that metabolism may influence test results independent of disease state.

Our findings demonstrate that an ECL-based P-tau217 bioassay can correctly identify people as amyloid positive in the A4/LEARN analysis set based on standardized measurements of amyloid such as PET, even with a relatively low CL threshold of 20 CL. Using this assay, we find that plasma P-tau217 is a viable and sensitive biomarker that is sufficient to predict amyloid PET. Performance of P-tau217 using this immunoassay was equivalent to recently published MS data from the AHEAD trial (34). We also found that P-tau217 demonstrated good pharmacodynamic biomarker properties in tracking change in amyloid PET over the trial with treatment. In addition to identification and tracking of change in amyloid PET status, we also found the bioassay to have excellent short-term repeatability correlation when using participant samples collected at different timepoints. Additionally, further investigation into the influence of other technical and biological variables on test results will help improve the overall utility of the bioassay. Ongoing work is specifically focused on how P-tau217 can predict tau PET and other tracers in the context of differential levels and regional distributions for amyloid and tau PET signal. Lastly, as alluded to above, one major priority of our current work involves expanding these findings to more representative populations (45) to determine whether specific plasma P-tau217 cutoff values exist and their relation to amyloid and tau PET status across different racial, ethnic groups.

### Electronic Supplementary Material

Supplementary material, approximately 4.63 MB.

## Notes

*Ethical Considerations:* Approval from an institutional review board or ethics committee was obtained at each of the sites. All participants and their study partners provided written informed consent prior to data collection, which included consent for data sharing.
