# Supplementary figures and images for: Longitudinal Phospho-tau217 Predicts Amyloid Positron Emission Tomography in Asymptomatic Alzheimer's Disease
Source: J Prev Alzheimers Dis. 2024 Jul 24;11(4):823–30. doi: 10.14283/jpad.2024.134 (PMC11266279; doi:10.14283/jpad.2024.134)

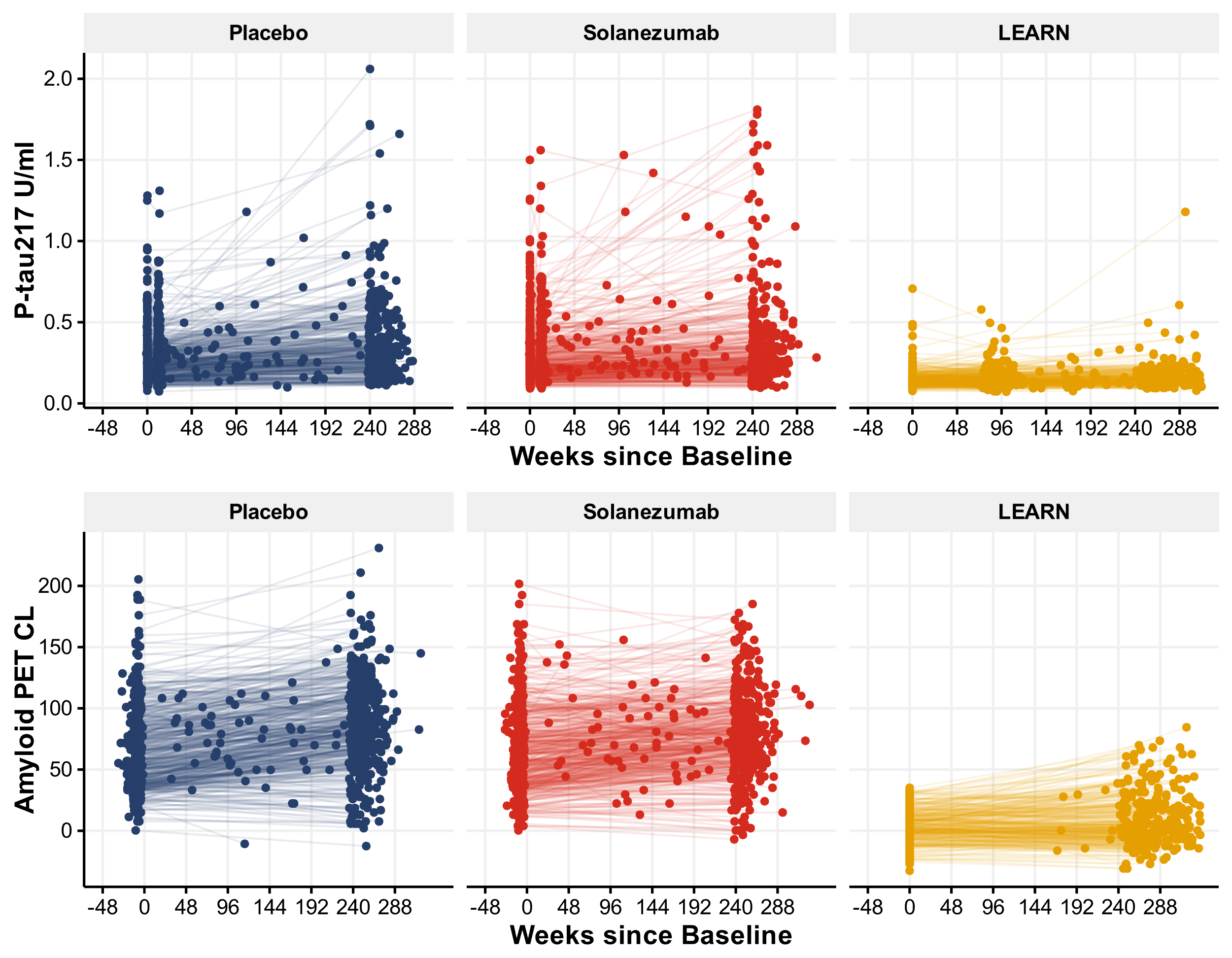


Figure S1: Spaghetti plots of P-tau217 and amyloid PET for A4 and LEARN

Supplement: Supplementary file 1 — Supplementary material, approximately 4.63 MB. [file mmc1.docx]
